# Mutational analysis in familial Alzheimer’s disease of Han Chinese in Taiwan with a predominant mutation *PSEN1* p.Met146Ile

**DOI:** 10.1038/s41598-020-76794-9

**Published:** 2020-11-13

**Authors:** Yung-Shuan Lin, Chih-Ya Cheng, Yi-Chu Liao, Chen-Jee Hong, Jong-Ling Fuh

**Affiliations:** 1 Department of Neurology, Neurological Institute, Taipei Veterans General Hospital, Taipei, 112 Taiwan; 2 Faculty of Medicine, National Yang-Ming University School of Medicine, Taipei, Taiwan; 3 Department of Psychiatry, Taipei Veterans General Hospital, Taipei, Taiwan; 4 Department of Pediatrics, Taipei Veterans General Hospital, Taipei, Taiwan; 5 Brain Research Center, National Yang-Ming University, Taipei, Taiwan

**Keywords:** Genetics, Neuroscience, Neurology

## Abstract

Mutations in *PSEN1*, *PSEN2*, or *APP* genes are known to be causative for autosomal dominant Alzheimer’s disease (ADAD). While more than 400 mutations were reported worldwide, predominantly *PSEN1*, over 40 mutations have been reported in Han Chinese and were associated with earlier onset and more affected family members. Between 2002 and 2018, 77 patients in the neurological clinic of Taipei Veterans General Hospital with a history suggestive of ADAD were referred for mutational analysis. We retrospectively collected demographics, initial symptoms, neurological features and inheritance. We identified 16 patients with *PSEN1* and 1 with *APP* mutation. Among the mutations identified, *PSEN1* p.Pro117Leu, p.Met146Ile, p.Gly206Asp, p.Gly209Glu, p.Glu280Lys and p.Leu286Val and *APP* p.Asp678His were known pathogenic mutations; *PSEN1* p.His131Arg and p.Arg157Ser were classified as likely pathogenic and variance of unknown significance respectively. The mean age at onset was 46.2 ± 6.2 years in patients with mutation found. *PSEN1* p.Met146Ile, occurred in 56.2% (9/16) of patients with *PSEN1* mutations, was the most frequent mutation in the cohort. The additional neurological features occurring in 9 *PSEN1* p.Met146Ile index patients were similar with the literature. We found patients with genetic diagnoses were more likely to have positive family history, younger age at onset and less brain white matter hyperintensity.

## Introduction

Alzheimer’s disease (AD) is the most common cause of dementia and is progressive and unremitting. While the majority of AD is late-onset and occurs sporadically, early-onset AD with autosomal dominant inheritance accounts for less than 1% of all AD cases^1^. AD is a neurodegenerative disorder that affects wide areas of the cerebral cortex and hippocampus^2^, and is associated with the accumulation of insoluble forms of amyloid­β (Aβ) in plaques outside neurons and aggregations of the microtubule protein tau in neurofibrillary tangles inside neurons. These changes are eventually accompanied by the damage and death of neurons. Aβ is derived from the proteolytic cleavage of amyloid precursor protein (*APP*, encoded by the *APP* gene) by a complex family of enzymes (γ­secretases and β­secretases). Presenilin 1, encoded by the *PSEN1* gene, and presenilin 2, encoded by the *PSEN2* gene, are the two major core proteins of γ-secretase^2^. Patients with autosomal dominant AD (ADAD) can be defined by mutations in the *PSEN1, PSEN2, or APP* genes and have ≥ 2 generations of AD starting younger than 65 years^3^. Among the 3 genes, *PSEN1 *is most frequently mutated (*PSEN1* > *APP* > *PSEN2*) and has the youngest onset age (30 to 50 years)^4^.

Except for younger age at onset (mean age of ~ 45 years), patients with ADAD usually have an amnestic presentation very similar to that seen in sporadic cases, with the initial symptom being a gradually worsening of the ability to remember new information. Atypical presentations other than cognitive dysfunction appear to be more common in ADAD^5^. For example, myoclonus and seizures are more frequent in familial cases. In Chinese Familial Alzheimer’s Disease Network involving 404 pedigree, 13.1% of pedigrees carried *PSEN1/PSEN2/APP* mutations. A total of 40 *PSEN1/PSEN2/APP* mutations were reported where 31 mutations were in *PSEN1*, 5 in *APP* and 4 in *PSEN2*^6^. Comparing with pedigrees of no mutations found, pedigrees with *PSEN1/PSEN2/APP* mutations had larger proportion of early-onset cases, more affected generation and family member and earlier pedigree mean AAO. In Taiwanese population, except a study on patients with AD (50 case and 50 control) in 2009 with 3 *APP* polymorphisms identified^7^, there were only sporadic case reports on familial AD with causative mutations found^8,9^. Han Chinese is the largest ethnic group with growing patients with AD. Despite genetic profile of ADAD been reported, the relationship between genotype and phenotype of Han Chinese AD, especially in Taiwan were lacking.

We aimed to study the genotypes and clinical phenotypes of ADAD among patients in the neurological clinic of Taipei Veterans General Hospital. We investigated the initial symptoms at onset, the frequency of presenting with cognitive symptoms, additional neurological features, and their associations with age at symptom onset and mutation position. The clinical manifestations of the individuals with specific mutations are also reported.

## Methods

### Participants

Between 2002 and 2018, 57 patients (25 males, 32 females) of the neurological clinic of Taipei Veterans General Hospital, a medical center in Taiwan, were referred for genetic analysis. We performed genetic testing in accordance with guideline from Matsuda et al.^10^ and Goldman et al.^11^. Genetic testing occurred in the context of genetic counseling (in person or in the presence of symptomatic patient’s family member). We used simple and easily comprehensible words to explain all matters and provided most recent and correct information to the subject. The participants were informed that there is no proven pharmacologic that reduces the risk of developing AD or stops its progression currently. Informed consent was obtained from all patients and their caregivers. This research project was approved by the institutional review boards at Taipei Veterans General Hospital. The inclusion criteria were (1) patients having at least one first-degree family member with impaired cognitive functions or early deceased parent, which suggested ADAD, or (2) sporadic AD patients with an age at onset (AAO) below age 65. Family pedigree of at least two generations was collected after informed consent was signed for genetic testing. Mutational analysis of the *PSEN1, PSEN2* and *APP* genes was performed in these 57 patients using the multiplex single-strand conformational polymorphism technique (SSCP) and subsequent Sanger sequencing. A portion of the relatives of the index patients with confirmed pathogenic mutations were counseled and received presymptomatic diagnosis with informed consent. Since 2018, we used next-generation sequencing (NGS) followed by Sanger sequencing to sequence all exons of *PSEN1, PSEN2* and *APP* genes in another 20 patients (8 males, 12 females) who met the inclusion criteria. We retrospectively collected these patients’ demographics, initial symptoms at onset and additional neurological features if available. A modified Goldman score^12^ was given to each patient according to their family pedigrees to indicate the inheritance pattern. A modified Goldman score of 1 is defined by the presence of at least three affected people in two generations, with one person being a first-degree relative of the other two; a score of 2 is familial aggregation of three or more family members with dementia not meeting the criteria for a score of 1; a score of 3 is one other affected family member with dementia (modified to give a score of 3 only if there is a history of young-onset dementia within the family, i.e., AAO less than 65 years; with a score of 3.5 if AAO is above 65); and a score of 4 is no or an unknown family history. We also performed visual rating analysis on the earliest available brain magnetic resonance imaging (MRI) of each patient (a total of 32 brain MRIs) performed at our hospital. The severity of medial temporal atrophy (MTA) was rated by the MTA score, ranging from 0 to 4 with regard to the width of the choroid fissure, the width of the temporal horn and the height of the hippocampal formation^13^. Posterior atrophy (PA) was rated from 0 to 3 regarding anatomical landmarks on axial/coronal/sagittal views^14^. White matter hyperintensities (WMHs) were rated on a 4-point scale (from 0 to 3, indicating normal to severe WMH)^15^.

### Next generation sequencing and variants filtering

Targeted regions were sequenced by the Illumina platform using a MiSeq sequencing system. The resulting sequencing reads were aligned to the human reference genome (GRCh37, hg19) using the BWA (Burrows–Wheeler Aligner) Enrichment v2.1.2 packages (BaseSpace, Illumina). The criteria of quality parameters of sequencing data obtained from the MiSeq sequencing system are listed below. The percentages of bases with a desirable quality score of Q30 were more than 90% in each sample. The mean region coverage depth was more than 800× and the percentages of target coverage at 50× were above 95%. The genome analysis toolkit (GATK) was used to conduct variants calling and analysis of disease related variants was carried out with the Variant Studio software v3.0.12 (Illumina). All variants obtained in this study passed the quality filters established in the above-mentioned software.

We filtered rare missense variants or splicing recognition sites by a list of candidate genes of dementia and by the following criteria: a. minor allele frequency less or equal to 0.01 in both gnomAD v.2.1.1 (Genome Aggregation Database)^16^ global and East Asian; b. not classified as benign/likely benign in ClinVar database^17^; c. rather evolutionary conserved defined by GERP++ (Genomic Evolutionary Rate Profiling++)^18^ score greater than 2; d. at least 2 out of 3 in silico prediction method, including CADD (Combined Annotation Dependent Depletion)^19^ score greater than 20, SIFT (Sorting Intolerant From Tolerant)^20^ and Polyphen-2 (Polymorphism Phenotyping v.2)^21^, suggested deleterious.

### Population and disease databases

We used 3 population databases including Exome Aggregation Consortium (ExAC)^22^, gnomAD v2.1.1 and Taiwan Biobank^23^ to check the frequency of the identified variants in the general population; and one disease database: Human Gene Mutation Database (HGMD)^24^, where variant annotations published in the literature were listed.

ExAC and gnomAD are databases of variants found during exome sequencing of 61,486 and 125,748 respectively, unrelated individuals sequenced as part of various disease-specific and population genetic studies. Taiwan Biobank, a nationwide research database formed to collect blood samples and biomedical information from 200,000 Taiwanese participants between 30 and 70 years of age using a population-based design^25^. It released whole-genome sequence data of 1517 unrelated Taiwanese people online (TaiwanView: taiwanview.twbiobank.org.tw/search).

### Statistical analysis

Continuous variables are expressed as the means ± standard deviations. Categorical variables are presented as numbers and ratios. We analyzed the modified Goldman score, AAO and additional neurological features of the index patients. Regarding these parameters, the Kolmogorov–Smirnov exam was used to confirm whether a normal distribution was followed; the Mann–Whitney analysis was used to examine whether there was any difference between patients carrying the *PSEN1* p.Met146Ile mutation and patients carrying *PSEN1* mutations other than p.Met146Ile. The following parameters were compared between the two groups: AAO; family history; and image characteristics of the MTA, PA and WMH. All statistical analyses were performed with SPSS software, version 18.0 (IBM Inc., Armonk, NY), with a *p* value < 0.05 defined as statistically significant.

### Ethics approval and consent to participate

Informed consent was obtained from all patients and their spouse or offspring who were legal representatives of the patient prior to participation in the study. This research project was approved by the institutional review boards at Taipei Veterans General Hospital.

### Consent for publication

Written informed consent was obtained from the patients for the publication of this research article. All authors have approved the manuscript for submission and gave consent for publication.

## Results

### AAO and family history

A total of 77 patients (33 male, 44 female) with mean age at onset of 51.1 ± 9.7 years underwent mutational analysis. There was no difference in the mean AAO between sexes (50.9 ± 9.7 years in male and 51.4 ± 9.8 years in female, *p* = 0.804). Using SSCP/Sanger sequencing, we identified 13 patients (8 males, 5 females) with a *PSEN1* mutation and 1 patient (female) with an *APP* mutation (see supplementary figure S1 for chromatogram). Subsequently, using the NGS technique, we found an additional 3 patients (1 male, 2 females) with a *PSEN1* mutation. We did not find any ADAD patients with *PSEN2* mutations in our cohort using either one of the two methods of genetic analysis. Compared with the 17 index cases carrying a pathological mutation and the other 60 patients with no mutations found, those with mutations had a younger AAO than those without mutations (mean AAO: 46.2 ± 6.2 years and 52.7 ± 10.0 years, respectively; *p* = 0.008). Patients with mutations in *PSEN1* or *APP* more frequently had a positive family history than those without any mutations (*p* = 0.001).

### Brain MRI findings

**Figure 1:**
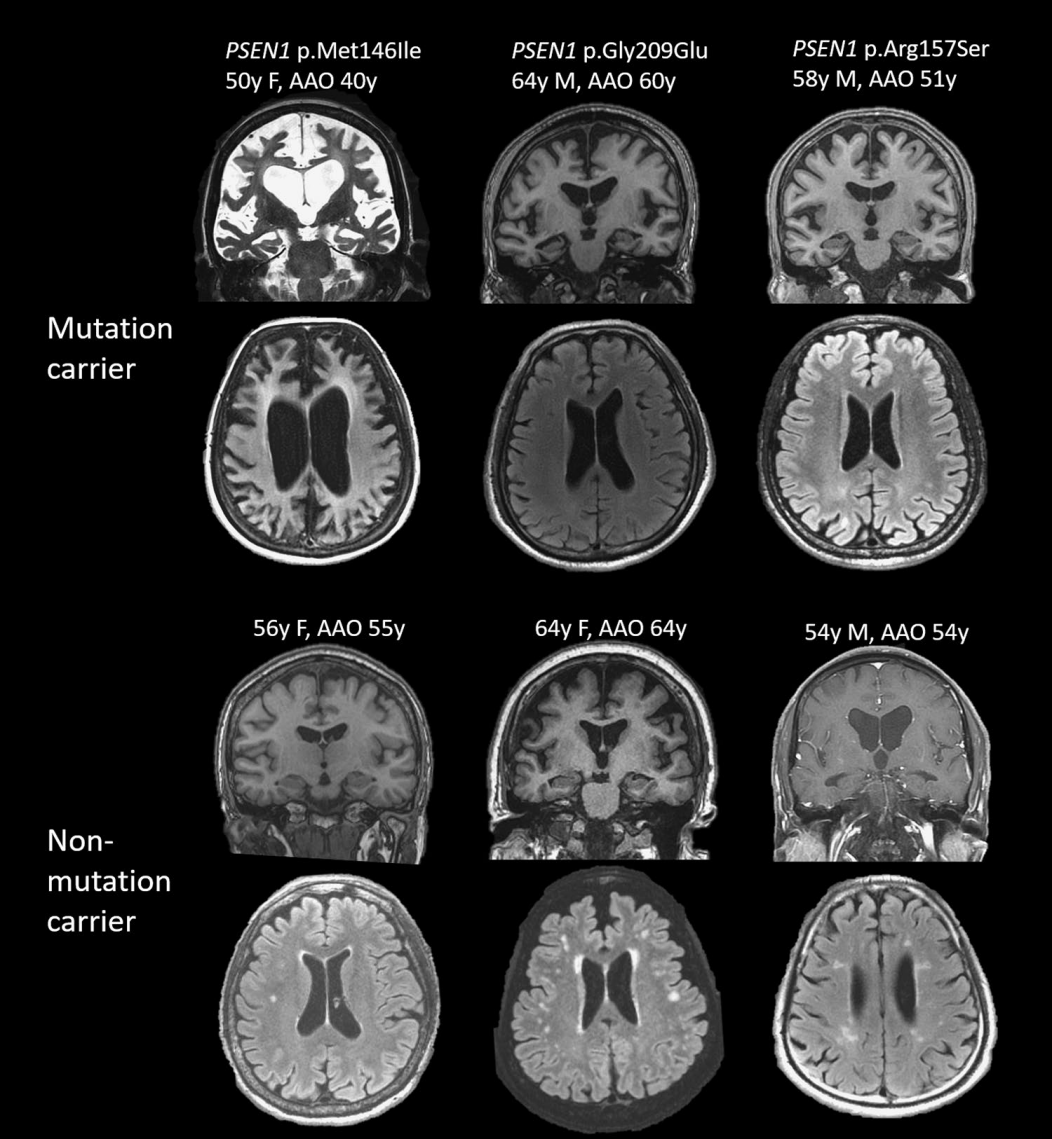
Representative MRI of each of 3 age-matched mutation carriers and non-carriers. Coded in the order of scale of medial temporal atrophy, posterior atrophy (PA) and white matter hyperintensity (WMH), from left to right in mutation carriers are (4,3,0), (2,1,0) and (1,2,0) and non-carriers are (1,1,1), (1,1,2) and (3,0,2). Various degree of MTA was noted across patients with or without mutation; in the contrary, WMH was seen predominantly in non-mutation carriers.

Brain MRI was available in 32 patients, including 11 with pathogenic mutations and 21 with no mutations found. The severity of MTA and PA did not differ between AD patients with and without mutations (*p* = 0.981 and *p* = 0.865, respectively). With adjustment of age, WMHs were significantly increased in those without mutations compared with those with mutations (B = 1.124, 95% CI 0.526–1.721, *p* < 0.001, VIF = 2.155) (Fig. 1).

### Brief history of patients with mutations

Among the *PSEN1* mutations identified in Taiwanese ADAD patients, the pathogenicity of the p.Pro117Leu, p.Met146Ile, p.Gly206Asp, p.Gly209Glu, p.Glu280Lys and p.Leu286Val mutations has been confirmed in AD patients of other ethnic backgrounds^26–32^, while two mutations (p.Arg157Ser and p.His131Arg) were mutations with limited literature reports (Table 1 and Supplementary table S1).

**Table 1:** Summary of mutations found in the cohort.

Gene	Exon	Nucleotide change	Protein change	Gnomad/ExAC (East Asian)/TWV	HGMD	PolyPhen-2	SIFT	CADD	No. of family recruited	Modified Goldman score^†^	Mean age at onset, years (range)	Additional neurological features (number of index patients)
*PSEN1*	5	c.350C > T	p.Pro117Leu	NA/NA/NA	Y	PD	D	29.6	1	3	36	Emotional liability
*PSEN1*	5	c.392A > G	p.His131Arg	NA/NA/NA	Y	B	D	23.3	1	1	49	Headache
*PSEN1*	5	c.438G > A	p.Met146Ile	NA/NA/NA	Y	PD	D	28.0	9	1–2	44.7 (40–48)	^‡^Myoclonus (3), seizure (2), ^§^EPS (1), emotional liability (1)
*PSEN1*	5	c.471G > T	p.Arg157Ser	NA/0.0009244/ 0.00033	NA	PD	D	31.0	1	4	51	Depression
*PSEN1*	7	c.617G > A	p.Gly206Asp	NA/NA/NA	Y	PD	D	28.4	1	3	33	Seizure
*PSEN1*	7	c.626G > A	p.Gly209Glu	NA/NA/NA	Y	PD	D	28.5	1	2	60	Apraxia
*PSEN1*	8	c.838G > A	p.Glu280Lys	NA/NA/NA	NA	PD	D	32.0	1	4	55	–
*PSEN1*	8	c.856C > G	p.Leu286Val	NA/NA/NA	Y	PD	D	27.5	1	1	43	–
*APP*	16	c.2066G > C	p.Asp678His	NA/NA/NA	Y	PD	T	26.2	1	4	50	–

gnomAD, Genome Aggregation Database; ExAC, Exome Aggregation Consortium; TWV, Taiwan View; HGMD, Human Gene Mutation Database; PolyPhen-2, Polymorphism Phenotyping v2; SIFT, Sorting Intolerant From Tolerant; CADD, Combined Annotation Dependent Depletion; NA, not applicable; PD, probably damaging; B, benign; D, deleterious; T, tolerated; Y, mutation listed in HGMD.

^**†**^A modified Goldman score of 1 is defined by the presence of at least three affected people in two generations, with one person being a first-degree relative of the other two; a score of 2 is familial aggregation of three or more family members with dementia not meeting the criteria for a score of 1; a score of 3 is one other affected family member with dementia (modified to give a score of 3 only if there is a history of young-onset dementia within the family, i.e., AAO less than 65 years; with a score of 3.5 if AAO is above 65); and a score of 4 is no or an unknown family history.

^‡^Numbers in parentheses indicate the number of index patients with additional neurological features.

^§^EPS extrapyramidal symptoms.

*PSEN1* c.350C > T, p.Pro117Leu was found in a male patient who had a progressive decline in memory since age 36, followed by myoclonic jerks. His father had poor memory before his premature death at age 42 due to a traffic accident.

**Figure 2:**
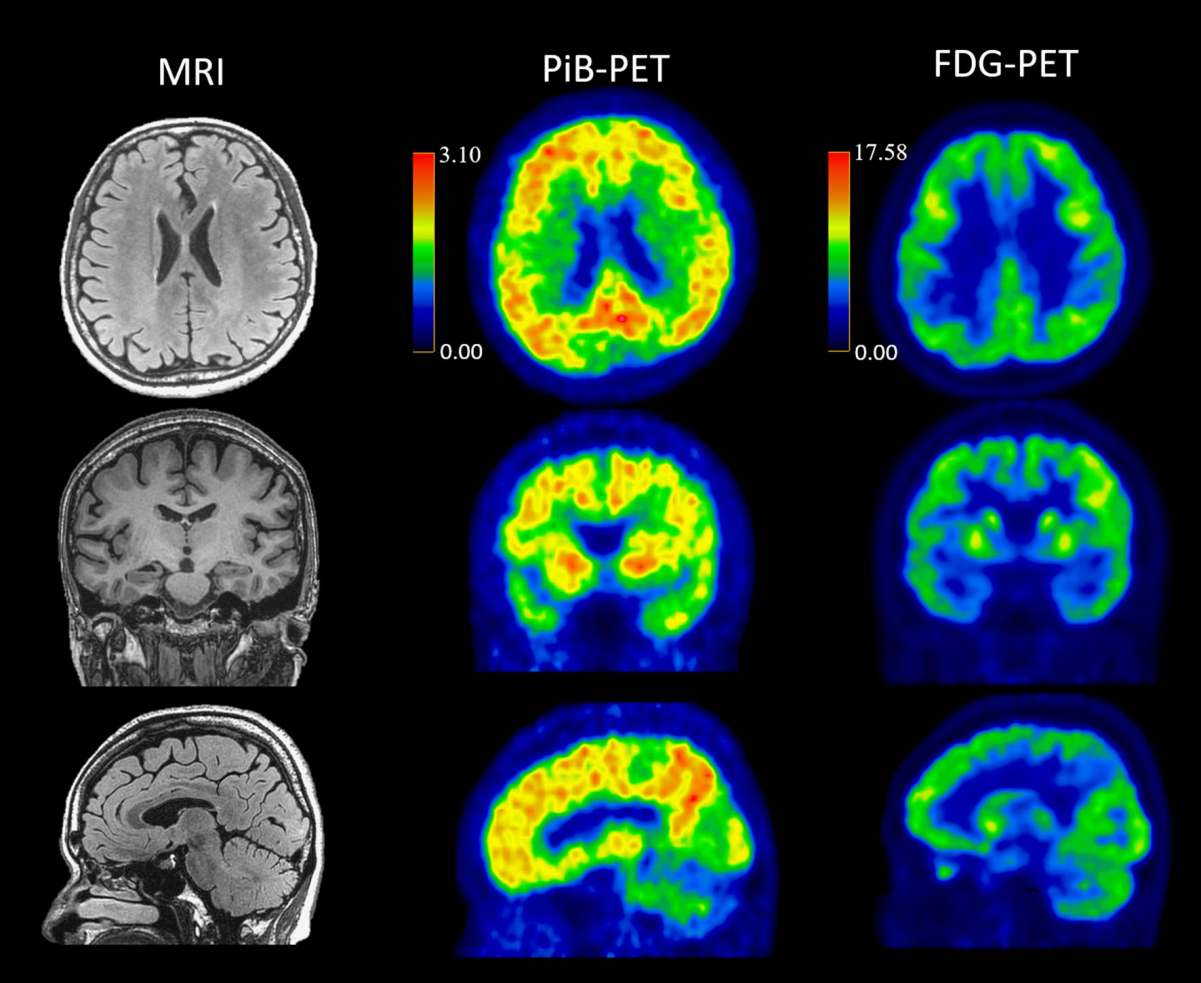
Brain imaging of a patient with *PSEN1* p.His131Arg and age at onset of 49 years. Brain MRI at age 61 and PiB-PET and FDG-PET at age 62 were showed. MRI showed mild medial temporal atrophy, mild parietal atrophy and minimal white matter hyperintensity; PiB-PET demonstrated extensive cortical and striatal amyloid deposition; FDG-PET showed decreased uptake in precuneus and bilateral temporo-parietal region.

*PSEN1* c.392A > G, p.His131Arg was discovered in a female patient who had impaired memory and topographic disorientation since age 49. Pittsburgh compound B positron emission tomography (PiB-PET) amyloid imaging of this patient demonstrated extensive cortical and striatal amyloid deposition (Fig. 2). Her elderly sister had amnesia at age 52, and her father died prematurely at age 65, supporting the autosomal dominant inheritance pattern.

*PSEN1* c.438 G > A, p.Met146Ile was discovered in 9 families, 6 of which had a very strong family history (modified Goldman score 1). Patients showed typical amnestic symptoms and had other neurological symptoms, such as seizures, myoclonic jerks, extrapyramidal symptoms (EPS) and emotional liability.

*PSEN1* c.471G > T, p.Arg157Ser was found in one male patient who had amnesic onset at age 51. A family history was not available. This mutation also had been reported in a Chinese male with AAO of 60 years and his mother died in the 10th decade with dementia^33^.

*PSEN1* c.617G > A, p.Gly206Asp was discovered in a female patient who showed memory impairment at age 33 and later had seizures. She was an adopted child, and her biological father died younger than 65 years of age with demented features.

*PSEN1* c.626G > A, p.Gly209Glu; *PSEN1* c.838G > A, p.Glu280Lys; and *PSEN1* c.856C > G, p.Leu286Val were found in one patient each, and the AAO in these patients was 60, 55, and 43, respectively. A positive family history was found in patients with p.Gly209Glu and p.Leu286Val.

The only *APP* gene mutation we identified was c.2066G > C, p.Asp678His in a female patient who had progressive dementia onset at age 50. The mother and maternal aunt of the index patient had memory impairment in their 7th decade. This mutation had been reported in another Taiwanese AD family in 2012^8^.

### ***PSEN1*** p.Met146Ile mutation vs ***PSEN1*** mutations other than p.Met146Ile mutation (Table 2)

**Table 2:** Number of the index patients with additional neurological features in the groups of *PSEN1* p.Met146Ile and *PSEN1* mutations other than p.Met146Ile^†^.

	*PSEN1* p.Met146Ile (n/9)	*PSEN1* mutations other than p.Met146Ile (n/7)
Myoclonus	3/9	1/7
Seizure	2/9	1/7
EPS^‡^	1/9	0/7
Emotional liability	1/9	1/7

EPS extrapyramidal symptoms.

^†^All additional neurological features are not significantly different between the two groups.

**Figure 3:**
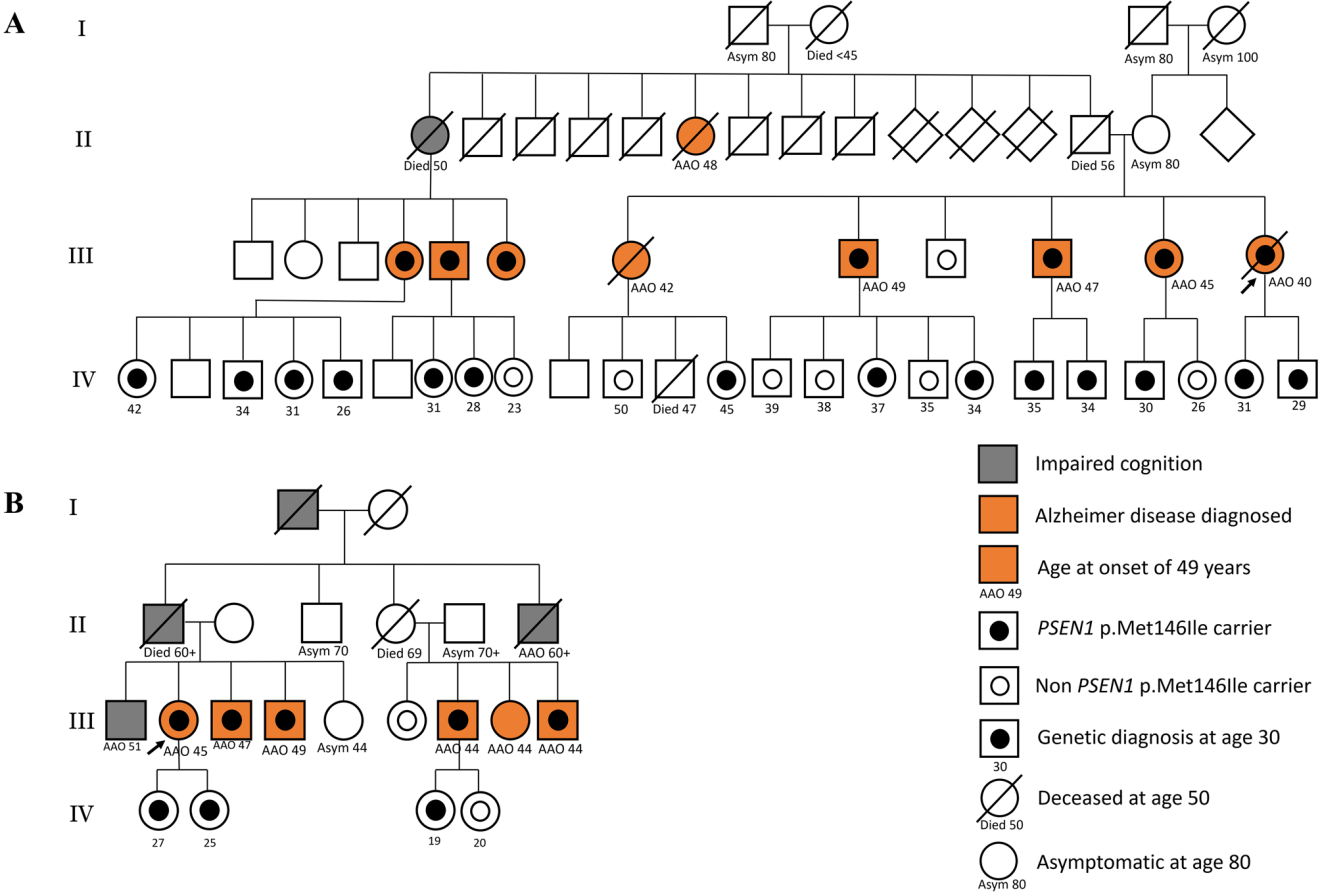
Two large pedigrees with four generations recruited demonstrating autosomal dominant inheritance of AD. Two representative family pedigrees of the cohort are demonstrated. Square indicates male and circle indicates female. Individuals with impaired cognition or diagnosed Alzheimer disease are given background color of gray or orange respectively. Inner circle filled with black indicates genetically confirmed *PSEN1* p.Met146Ile carrier whereas hollow inner circle indicates non *PSEN1* p.Met146Ile carrier. Square and circle symbols without inner circle indicate individuals without genetic tests.

Among the 16 index AD patients with *PSEN1* pathogenic mutations, the *PSEN1* c.438G > A, p.Met146Ile mutation was found in nine patients and accounted for 56.2% (9/16) of patients with *PSEN1* mutations. The 9 index patients with *PSEN1* p.Met146Ile were unrelated according to their self-provided pedigree. We compared the clinical features between patients with *PSEN1* p.Met146Ile mutation and those with other *PSEN1* mutations. The modified Goldman score, AAO and presence of additional neurological features did not follow a normal distribution when using Kolmogorov–Smirnov examination. Using Mann–Whitney analysis, the Goldman score was not different between the patients with p.Met146Ile mutation and those with *PSEN1* mutations other than p.Met146Ile (*p* = 0.148); the AAO between the p.Met146Ile and *PSEN1* mutations other than p.Met146Ile groups was similar (*p* = 0.457), and so was the years of education (*p* = 0.521). The distribution of other neurological features, including myoclonus, seizures, EPS and emotional liability, was also similar between the two groups. From the nine index AD patients with *PSEN1* p.Met146Ile mutation, we were able to recruit two large pedigrees. Family one (Fig. 3a) had 10 subjects with clinically diagnosed or suspected AD, 3 premature deaths and 21 subjects with genetically confirmed mutations among the youngest 2 generations. Family two (Fig. 3b) had 10 subjects with clinically diagnosed or suspected AD and 8 subjects with genetically confirmed mutations. Both families had a modified Goldman score of 1, and among the symptomatic families with confirmed mutations, the AAO was in their 40s. The oldest asymptomatic *PSEN1* p.Met146Ile carrier in families one and two was aged 45 and 27, respectively, at the time of genetic analysis.

## Discussion

This is a study performing extensive mutational analysis in Taiwanese familial AD (FAD). In our cohort, most patients carried *PSEN1* mutations, in which p.Met146Ile was the most frequent mutation.

Over 300 mutations in *PSEN1* have been reported in the literature, in which close to 270 were pathogenic. Until 2019, 57 mutations of *PSEN1* were discovered in Asian countries, including Japan, Korea, the People’s Republic of China, Iran and Thailand^34^. A recently published systemic review disclosed that a *PSEN1* mutation was found in 66.7% of Chinese FAD and 72.7% of Asian (Chinese, Korean, and Japanese) FAD families^35^. Our study showed similar findings that most of the ADAD-associated mutations were detected in *PSEN1.* Furthermore, the *PSEN1* p.Met146Ile mutation is the most frequent mutation and occurred in more than half of our ADAD patients carrying *PSEN1* mutations. The index patients were unrelated according to their self-reporting family pedigree. While *PSEN1* p.Met146Ile has not been reported in Taiwanese or Asian populations before, it was first reported in 1996 in a Danish family and cosegregates with AD with an age of onset in the early forties^28^, and later found in a British family with mean age at onset of 49 years^36^. In a referral-based series of AD cases which included 372 patients with AD and 42 asymptomatic individuals with a strong family history of AD. *PSEN1* p.Met146Ile (c.438G > T) and p.Met146Leu (c.436A > T and c.436A > C) was each identified in one patient^31^. Among them, evidence for cosegregation with AD was available for p.Met146Leu in one family where there were multiple living affected members. A Swedish family with six cases of documented dementia across four consecutive generations was first described in 1946 and genetic analysis was performed in 1998 which disclosed *PSEN1* c.438G > C, p.Met146Ile mutation^37,38^. Cognitive decline started between 35 and 49 years of age in all 6 affected cases who later developed typical symptoms of AD with myoclonic twitching and seizures. In presenilin 1, amino acid residue Met146 is fully conserved in most animal presenilins and is included in a hydrophobic α-helical structure, which is a putative transmembrane domain. Mutations that replace a hydrophobic methionine with another hydrophobic amino acid (leucine or isoleucine), eg. p.Met146Ile (G > T or G > C) and p.Met146Leu (A > T or A > C), were characterized as pathogenic mutations rather than neutral polymorphisms.

The *PSEN1* p.His131Arg mutation was first reported as a variance of unknown significance by Ikeda et al. in 2013^39^, where a Japanese female patient had amnesia onset at age 45 with a negative family history. This variant was absent from the variant databases. In silico analyses predicted the p.His131Arg substitution to be benign by Polyphen-2, but damaging by SIFT. Using this mutant, Aβ42/Aβ40 ratio was slightly increased in an in vitro assay but in mouse neuroblastoma cells, there was an approximately twofold increase^40^. Therefore, the researcher described the originally variance of unknown significance to be likely pathogenic in 2020. With the positive PiB-PET of our patient, solid clinical evidence was added to *PSEN1* p.His131Arg.

*PSEN1* p.Arg157Ser was found in population database with low minor allele frequency where ExAC east Asian of 0.0009244 and Taiwan Biobank of 0.00033 and bioinformatics tools, including PolyPhen-2, SIFT and CADD, all predicted this mutation to have a deleterious effect. Because of limited access to family data in both our patient and the reported Chinese patient, *PSEN1* p.Arg157Ser was classified as variance of unknown significance.

Pathogenic *APP* mutations are located mostly in exons 16 and 17^41^. The amino acid residue Asp678 is encoded by exon 16 of *APP*, and two different mutations at this residue have been previously reported in another Taiwanese AD family (p.Asp678His, in 2014) and in Japan (p.Asp678Asn). Mutations in the 670th to 682nd codon have been reported in Western countries. In the literature review, we found two more Taiwanese families located both in southern and northern Taiwan^8,42^. Combining previous reports and our findings, *APP* p.Asp678His seems to be the most common mutation in *APP* in the Taiwanese population.

Aside from having the earliest AAO, previous studies have found that patients with *PSEN1* mutations are more commonly affected by myoclonus (47%), seizures (24%), pyramidal signs (25%), EPS (14%), and cerebellar signs (4%)^1,43^. In our index patients with *PSEN1* p.Met146Ile mutation, myoclonus, seizure and EPS occur in 33.3%, 22.2% and 11.1% respectively. Our result was similar with previous studies.

Regarding patient selection for genetic analysis for FAD, our study revealed that those with genetic diagnoses were more likely to have a positive family history, a younger age at onset and less WMH. Thus, careful selection of patients with the aforementioned characteristics is more likely to have positive findings in genetic analysis. WMHs, which are known to be associated with traditional vascular risk factors such as hypertension, diabetes and smoking, triple the risk of stroke and double the risk of cognitive impairment and dementia^44^. We attributed our result of lesser degree in WMH in ADAD patients to younger age with presumably lower incidence of vascular risk factors. However, there was study reported greater total WMH volumes which appear before the expected symptom onset in mutation carriers from DIAN study^45^. The incongruous results might come from different method to measure WMH (visual 4-point scale versus voxel-based quantification) and need more studies to clarify.

In concordance with the literature, *PSEN1/PSEN2/APP* only explained a small portion (17/77, 22%) of early onset familial AD in our cohort. For those genetically unexplained, the role of other neurodegenerative brain diseases genes and C7, a novel risk gene identified in Han Chinese^46^, in familial and early-onset AD expression should be considered^47^.

Genetic analysis and subsequent counseling do not alter the clinical management of patients with early-onset AD but may help with future life planning or, more specifically, reproductive planning. Unaffected individuals who request presymptomatic diagnosis should be offered the information and additional support because there is no curative treatment for AD if they test positive^48^. The identification of mutation carriers in FAD patients in an asymptomatic state may contribute to the understanding of the progress of AD from preclinical to various clinical stages.

Our study had several limitations: first, limited numbers of affected families; second, a retrospective design; third, haplotype analysis may be helpful to confirm the index patients were genetically unrelated. Nonetheless, we hereby report an extensive mutational analysis of a Taiwanese FAD cohort with 17 out of 77 index patients carrying either *PSEN1* or *APP* gene mutation. In addition to the most prevalent mutation *PSEN1* p.Met146Ile among the cohort, we also report a likely pathogenic mutation of *PSEN1* p.His131Arg, which is previously a variance of unknown significance. Patients with genetic diagnoses were more likely to have positive family history, younger age at onset and less brain white matter hyperintensity, comparing with those without causative mutations found.

## Supplementary information

Supplementary Figure.

Supplementary Table.

## Data Availability

The datasets used and/or analyzed during the current study are available from the corresponding author upon request.

## Abbreviations

AD: Alzheimer’s disease
ADAD: Autosomal dominant Alzheimer’s disease
AAO: Age at onset
NGS: Next-generation sequencing
MRI: Magnetic resonance imaging
MTA: Medial temporal atrophy
PA: Posterior atrophy
WMH: White matter hyperintensity
PiB-PET: Pittsburgh compound B positron emission tomography
PolyPhen-2: Polymorphism Phenotyping v2
SIFT: Sorting Intolerant From Tolerant
CADD: Combined Annotation Dependent Depletion
EPS: Extrapyramidal symptoms
FAD: Familial AD
