# Supplementary material for: Mutational analysis in familial Alzheimer’s disease of Han Chinese in Taiwan with a predominant mutation PSEN1 p.Met146Ile
Source: Sci Rep. 2020 Nov 13;10:19769. doi: 10.1038/s41598-020-76794-9 (PMC7666133; doi:10.1038/s41598-020-76794-9)
Supplement: Supplementary file 1 — Supplementary Figure. [file 41598_2020_76794_MOESM1_ESM.pdf]

Mutational analysis in familial Alzheimer's disease of Han Chinese in Taiwan with a predominant mutation *PSEN1* p.Met146Ile

Yung-Shuan Lin, MD[1,2], Chih-Ya Cheng, PhD[3,4], Yi-Chu Liao, MD, PhD[1,2], Chen-Jee Hong, MD [2,3*], Jong-Ling Fuh, MD [1,2,5*]

[1]Department of Neurology, Neurological Institute, Taipei Veterans General Hospital, Taipei, Taiwan, [2]Faculty of Medicine, National Yang-Ming University School of Medicine, Taipei, Taiwan, [3]Department of Psychiatry, Taipei Veterans General Hospital, Taipei, Taiwan, [4]Department of Pediatrics, Taipei Veterans General Hospital, Taipei, Taiwan, [5]Brain Research Center, National Yang-Ming University, Taipei, Taiwan

*These authors contributed equally to the manuscript
First author: Dr. Yung-Shuan Lin (E-mail: yslin31@vghtpe.gov.tw)
Corresponding author: Dr. Jong-Ling Fuh (E-mail: jlfuh@vghtpe.gov.tw)
Co-corresponding author: Dr. Chen-Jee Hong (E-mail: cjhong@vghtpe.gov.tw)
Address: Department of Neurology, Neurological Institute, Taipei Veterans General Hospital, Taipei, Taiwan, 112
TEL: 886-2-28762522; FAX: 886-2-28765215

Supplementary figure S1. Chromatogram of one single-strand conformational polymorphism and Sanger sequencing of each index patient's mutation.

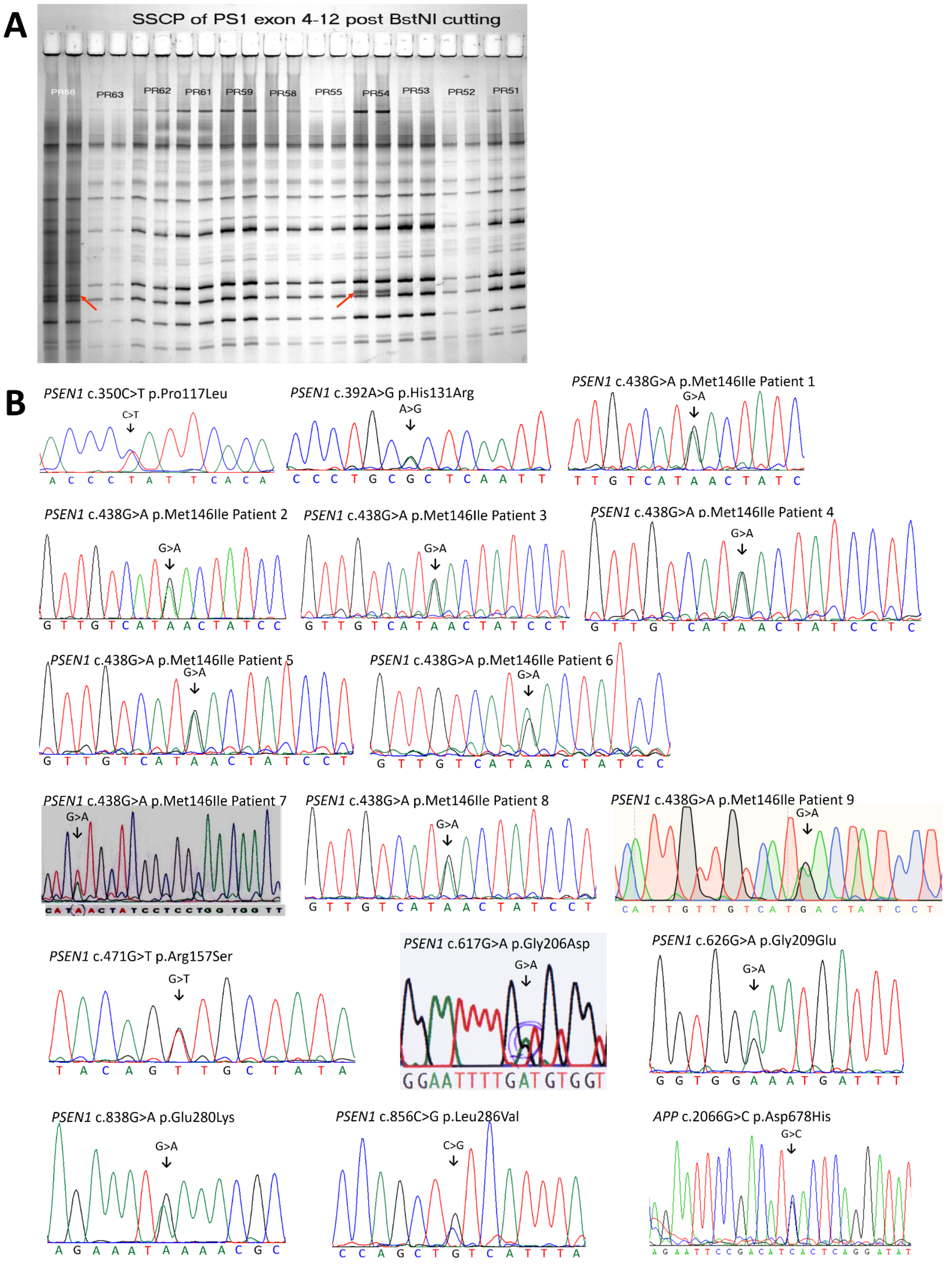

Panel A shows a representative result of the single-strand conformational polymorphism (SSCP) technology. The red arrows show variant bands in a chromatogram of single-strand conformational polymorphism, indicating possible variation in DNA fragments. All the samples were duplicated. Panel B displays the chromatograms of all mutations of *PSEN1* and *APP* gene found in the study.
