# Supplementary material for: Mutational analysis in familial Alzheimer’s disease of Han Chinese in Taiwan with a predominant mutation PSEN1 p.Met146Ile
Source: Sci Rep. 2020 Nov 13;10:19769. doi: 10.1038/s41598-020-76794-9 (PMC7666133; doi:10.1038/s41598-020-76794-9)
Supplement: Supplementary file 2 — Supplementary Table. [file 41598_2020_76794_MOESM2_ESM.pdf]

Mutational analysis in familial Alzheimer's disease of Han Chinese in Taiwan with a predominant mutation *PSEN1* p.Met146Ile

Yung-Shuan Lin, MD[1,2], Chih-Ya Cheng, PhD[3,4], Yi-Chu Liao, MD, PhD[1,2], Chen-Jee Hong, MD [2,3*], Jong-Ling Fuh, MD [1,2,5*]

[1]Department of Neurology, Neurological Institute, Taipei Veterans General Hospital, Taipei, Taiwan, [2]Faculty of Medicine, National Yang-Ming University School of Medicine, Taipei, Taiwan, [3]Department of Psychiatry, Taipei Veterans General Hospital, Taipei, Taiwan, [4]Department of Pediatrics, Taipei Veterans General Hospital, Taipei, Taiwan, [5]Brain Research Center, National Yang-Ming University, Taipei, Taiwan

*These authors contributed equally to the manuscript
First author: Dr. Yung-Shuan Lin (E-mail: yslin31@vghtpe.gov.tw)
Corresponding author: Dr. Jong-Ling Fuh (E-mail: jlfuh@vghtpe.gov.tw)
Co-corresponding author: Dr. Chen-Jee Hong (E-mail: cjhong@vghtpe.gov.tw)
Address: Department of Neurology, Neurological Institute, Taipei Veterans General Hospital, Taipei, Taiwan, 112
TEL: 886-2-28762522; FAX: 886-2-28765215

Supplementary table S1. Mutational and clinical information of the index patients

No.	Gene	Exon	Nucleotide	Amino acid	Method	AAO (year)	mGS[†]	Sex	Initial symptoms	Additional neurological feature	Age of MRI (year)	MTA	PA	WMH
1	*PSEN1*	5	c.350C>T	p.Pro117Leu	SSCP	36	3	M	Memory	Myoclonus<br>Emotional liability	38	0	0	0
2	*PSEN1*	5	c.392A>G	p.His131Arg	NGS	49	1	M	Memory<br>Topo[‡]	-	61	2	1	1
3	*PSEN1*	5	c.471G>T	p.Arg157Ser	NGS	51	4	M	Memory	-	59	1	2	0
4	*PSEN1*	5	c.438G>A	p.Met146Ile	SSCP	45	1	F	Memory	-	-	-	-	-
5	*PSEN1*	5	c.438G>A	p.Met146Ile	SSCP	45	1	F	Topo	-	45	-	0	1
6	*PSEN1*	5	c.438G>A	p.Met146Ile	SSCP	40	1	F	Memory	Seizure<br>EPS[§]	47	-	3	0
7	*PSEN1*	5	c.438G>A	p.Met146Ile	SSCP	48	1	M	Memory	-	48	1	1	0
8	*PSEN1*	5	c.438G>A	p.Met146Ile	SSCP	46	1	M	Memory	-	-	-	-	-
9	*PSEN1*	5	c.438G>A	p.Met146Ile	SSCP	40	1	F	Memory<br>Topo	Myoclonus<br>Emotional liability	46	1	0	0
10	*PSEN1*	5	c.438G>A	p.Met146Ile	SSCP	43	2	M	Language<br>Dysexecutive	-	46	1	2	0
11	*PSEN1*	5	c.438G>A	p.Met146Ile	SSCP	46	2	M	Memory	Myoclonus	46	0	0	0
12	*PSEN1*	5	c.438G>A	p.Met146Ile	SSCP	44	1	M	Memory	Myoclonus<br>Seizure<br>Choreoathetosis	55	2	1	0

										Auditory hallucination				
13	*PSEN1*	7	c.617G>A	p.Gly206Asp	SSCP	33	3	F	Memory	Seizure	-	-	-	-
14	*PSEN1*	7	c.626G>A	p.Gly209Glu	SSCP	60	2	M	Memory	Apraxia	64	2	1	0
15	*PSEN1*	8	c.838G>A	p.Glu280Lys	NGS	55	4	F	Memory	-	-	-	-	-
16	*PSEN1*	8	c.856C>G	p.Leu286Val	SSCP	43	1	M	Memory	-	-	-	-	-
17	*APP*	16	c.2066G>C	p.Asp678His	SSCP	50	4	F	Memory	-	-	-	-	-

mGS, modified Goldman score; AAO, age at onset; MTA, medial temporal atrophy; PA, posterior atrophy; WMH, white matter hyperintensity; SSCP, single strand-conformational polymorphism with subsequent Sanger sequencing; NGS, next generation sequencing

† A modified Goldman score of 1 is defined by the presence of at least three affected people in two generations, with one person being a first-degree relative of the other two; a score of 2 is familial aggregation of three or more family members with dementia not meeting the criteria for a score of 1; a score of 3 is one other affected family member with dementia (modified to give a score of 3 only if there is a history of young-onset dementia within the family, i.e., AAO less than 65 years; with a score of 3.5 if AAO is above 65); and a score of 4 is no or an unknown family history.

‡Topo, topographic disorientation

§EPS, extrapyramidal symptoms
